# Patients with longstanding psoriatic arthritis can achieve DAPSA remission or low disease activity and it correlates to better functional outcomes: results from a Latin-American real-life cohort

**DOI:** 10.1186/s42358-023-00338-8

**Published:** 2024-01-02

**Authors:** Larissa Vargas Cruz, Júlia Boechat Farani, Júlia Rabello Costa, João Victor de Andrade Águas, Bruna Ruschel, Franciele de Almeida Menegat, Andrese Aline Gasparin, Claiton Viegas Brenol, Charles Lubianca Kohem, Adrieli Bessa, Francisco Forestiero, Felipe Thies, Penélope Esther Palominos

**Affiliations:** 1 Hospital de Clínicas de Porto Alegre, Porto Alegre, Rio Grande do Sul Brazil; 2 Universidade Federal do Rio Grande do Sul, Porto Alegre, Rio Grande do Sul Brazil; 3 Novartis Biociências, Porto Alegre, Brazil

**Keywords:** Arthritis, Psoriatic, Disease activity, Disability evaluation, Patient reported outcome measures, Function

## Abstract

**Background:**

Patients with psoriatic arthritis (PsA) experience reduced physical function and impaired quality of life. Better patient-reported functional outcomes are found when lower disease activity is achieved.

**Objectives:**

To evaluate the variation of physical function by HAQ-DI over time in PsA patients treated with standard therapy in a real-life setting: to verify predictors of achieving a minimum clinically important difference (MCID) in function by HAQ-DI (ΔHAQ-DI ≤ − 0.35) and to measure the impact of achieving REM/LDA on long-term function by HAQ-DI.

**Methods:**

This is a longitudinal analysis of a real-life retrospective cohort. Data from PsA patients with at least 4 years of follow-up in the PsA clinic from 2011 to 2019 were extracted from electronic medical records. The variations of physical function by HAQ-DI and disease activity by DAPSA over time were calculated. A multivariate hierarchical regression model was applied to verify predictors of MCID in HAQ-DI. A comparison of HAQ-DI variation between patients with DAPSA REM, LDA, moderate and high disease activity was made using the generalized estimating equation model (GEE), adjusted by Bonferroni test. The Spearman correlation method was applied to verify the correlation of ΔDAPSA and ΔHAQ-DI over time. Statistical analysis was performed in SPSS program version 21.0.

**Results:**

Seventy-three patients were included in the analysis. Physical function measured by HAQ-DI was determined by PsA disease activity measured by DAPSA (*p* < 0.000). A moderate and statistically significant correlation between ΔDAPSA and ΔHAQ-DI was observed (r_s_ = 0.60; *p* < 0.001). Only patients in DAPSA REM demonstrated a constant decline in HAQ-DI scores during the follow-up. White ethnicity and older age at baseline were predictors for not achieving MCID in HAQ-DI [RR 0.33 (0.16–0.6795% CI *p* = 0.002) and RR 0.96 (0.93–0.9895% CI *p* < 0.000), respectively, while higher scores of HAQ-DI at baseline were predictors of achieving MCID [RR 1.71 (1.12–2.6095%CI *p* = 0.013)].

**Conclusions:**

In PsA, patients who maintained DAPSA REM/LDA over time had better long-term functional outcomes. Higher HAQ-DI scores at baseline, non-white ethnicity and younger age were predictors for achieving a clinical meaningful improvement of HAQ-DI.

## Background

Psoriatic arthritis (PsA) is a chronic inflammatory arthropathy associated with psoriasis, with an estimated prevalence of 133 every 100,000 subjects worldwide [1]. Poor disease control can lead to severe functional impairment and reduced health-related quality of life, with a significant global health burden [2]. Besides that, lower disease activity leads to better patient-reported functional outcomes [3–5].Therefore, it is widely recommended that the treatment goals for PsA should be remission or, alternatively, low to minimal disease activity, although there is no consensus on the best tool to assess or define remission [6–8]. In that sense, measurement of function and its correlation to disease activity in PsA constitute a major concern in Rheumatology.

To quantify disease activity in PsA, Disease Activity Index for PsA (DAPSA) and Minimal Disease Activity criteria (MDA) are the main tools currently used by Rheumatologists in daily practice [9, 10]. As for the assessment of physical function, patient-reported Health Assessment Questionnaire-Disability Index (HAQ-DI) is proven to be a valuable measure in PsA. Despite originally being developed for use in patients with rheumatoid arthritis [11–13], HAQ-DI has been validated and widely used in PsA clinical trials, even showing correlation to the effectiveness of active therapies when compared to placebo [14].

Clinical data correlating disease activity and function were mainly obtained in randomized clinical trials (RCTs), in which patients are followed in a controlled environment, have less comorbidities, a moderate to high disease activity at baseline and are usually treated with immunobiological disease-modifying antirheumatic drugs (bDMARDs) [3–5, 15, 16]. In such context, a major improvement in HAQ-DI scores over time is expected. However, those results might not be applicable in low and middle income countries, where patients have poor access to bDMARDs and commonly have long-term PsA, chronic deformities and lower rates of adherence to therapy.

The primary objective of this study was to evaluate the variation of physical function by HAQ-DI over time in PsA patients treated with standard therapy in a real-life setting. Secondary objectives were to quantify patients achieving a minimum clinically important difference (MCID) in function by HAQ-DI (ΔHAQ-DI ≤ − 0.35) [17]; to verify predictors of achieving a MCID in HAQ-DI; to quantify patients achieving sustained DAPSA remission (REM) or low disease activity (LDA) and patients achieving sustained MDA; and to measure the impact of achieving DAPSA REM or LDA (REM/LDA) on long-term function.

## Methods

### Study design

This is a longitudinal analysis of a real-life retrospective cohort of patients followed in Hospital de Clínicas de Porto Alegre’s PsA clinic from 2011 to 2019. Inclusion criteria were ≥ 18-year-old patients fulfilling the Classification Criteria for Psoriatic Arthritis (CASPAR) [18] with at least 4 years of follow-up in electronic medical records and at least two visits with HAQ-DI registered measurements within a 12-month interval. Data previously collected and registered in routine patient care from January 2011 to March 2019 were extracted from electronic medical records between August and October 2019. All data have been anonymized before analysis.

### Data collection

Demographics and clinical data collected at baseline were: patient age (years), gender, ethnicity (white or non-white), year of PsA diagnosis and year of PsA symptoms onset. Clinical data collected at baseline and at each subsequent visit were: patient global visual analogue scale (VAS) (0–10 cm); patient pain VAS (0–10 cm); patient fatigue VAS (0–10 cm); physician and patient skin VAS (0–10 cm); tender entheseal points with the Maastrich Ankylosing Spondylitis Enthesitis Score (MASES) (0–13); tender joint count (TJC) (0–68), swollen joint count (SJC) (0–66); serum C-reactive protein (CPR) level in mg/dL; assessment of disease activity by DAPSA LDA and LDA + REM and MDA; assessment of physical function by HAQ-DI score (0–3).

Disease activity by DAPSA was calculated by the sum of TJC (0–68), SJC (0–66), patient global VAS (0–10), patient pain VAS (0–10) and serum CRP (mg/dL). Remission (REM) was defined by DAPSA 0–4 and low disease activity (LDA)was defined by DAPSA 5–14 [9]. It was also recorded, at each consultation, if the patient achieved the state of Minimal Disease Activity (MDA), fulfilling 5 or more of the 7 following criteria: TJC ≤ 1, SJC ≤ 1, psoriasis body surface area (BSA) ≤ 3%, patient pain VAS ≤ 1.5, patient global VAS ≤ 2, tender entheseal points ≤ 1 and HAQ-DI ≤ 0,5 [10]. When the longitudinal analysis was performed, sustained REM/LDA by DAPSA and sustained MDA were defined as maintenance of REM/LDA or MDA over a period of >  = 12 months during follow-up.

Treatment data were also retrieved in each patient visit, including the use of synthetical disease-modifying anti-rheumatic drugs (sDMARDs): methotrexate, sulfassalazine, leflunomide;and the bDMARDs available at the time of study conduction: adalimumab, etanercept, infliximab, golimumab, ustekinumab and secukinumab. Patients were receiving standard care for PsA according to current national and international guidelines and recommendations [6–8].

### Statistical analysis

Sample size calculation was performed using WinPEP version 11.43 and was based on studies of Mease P. et al. (2018) [2] and Strand V. et al. (2018) [15]. Considering a 5% significance level, 90% power, an expected variation of 0.3 points on HAQ-DI in 4 years and an estimated standard deviation (SD) of 0.5 points, a minimal sample size of 43 patients was required. Quantitative variables were analyzed as mean and SD if normally distributed or median and interquartile range (25–75%) if they presented a non-normal distribution. Categorical variables were described as absolute numbers (n) and relative frequencies (percentage or %).

To evaluate the variation of physical function by HAQ-DI over time, we calculated the difference between median HAQ-DI at baseline and median HAQ-DI at the final patient visit (ΔHAQ-DI). To quantify patients achieving a MCID in HAQ-DI, we calculated the relative frequency (%) of patients with a ΔHAQ-DI ≤ − 0.35.

To verify which variables were predictors of achieving MCID in HAQ-DI, a multivariate hierarchical regression model was applied. Firstly, patients were divided in two groups according to achieving or not a MCID in HAQ-DI and the univariate analysis was performed. Then, variables with a p value of < 0.20 in this regression were selected for a second multivariate analysis, for which the significance level adopted was 5% (*p* < 0.05).

To quantify patients achieving DAPSA REM/LDA and MDA over time, we calculated the relative frequencies (%) of sustained REM/LDA and sustained MDA. To measure the impact of achieving DAPSA REM/LDA on long-term function by HAQ-DI, we performed a longitudinal analysis of median DAPSA and median HAQ-DI at baseline and at years 1, 2, 3, 4, 5 and 6, and performed a comparison of HAQ-DI variation between patients with DAPSA REM (= < 4), LDA (4–14), moderate (14–28) and high disease activity (> 28), using the generalized estimating equation model (GEE), adjusted by Bonferroni test. Also, the variation of median DAPSA at baseline and at the final patient visit was calculated (ΔDAPSA) and the Spearman correlation method was applied to verify the correlation of ΔDAPSA and ΔHAQ-DI over time.

Statistical analysis was performed in SPSS program version 21.0.

### Ethical considerations

The study followed international clinical research standards and was approved by the local ethics committee. Researchers signed a confidentiality term for the use of electronic data. The written informed consent from patients was not required because it was a retrospective study with data from routine care. Novartis collaborated with the study, but did not participate in the data collection, analysis or description of the results.

## Results

Seventy-three patients were included in the analysis, of which 58.9% (*n* = 43) were women and 89% (*n* = 65) had white ethnicity. At baseline, the mean (SD) age was 54.3 (9.9) years old, the median time (25–75th) of diagnosis of PsA was 8 (3–15) years and there was a median (25–75th) of 12 (5–20) years since the onset of articular symptoms until the baseline visit. Mean (SD) follow-up time was 6.2 (1.2) years. Mean (SD) number of patient visits with HAQ-DI registered measurements was 11.58 (4.02), corresponding to a mean of 1.86 visits per year. Further baseline and longitudinal data are described in Table 1**.**

**Table 1:** Characteristics of the 73 patients with PsA included in the analysis and univariate analysis of predictive factors to achieving a clinically meaningful improvement in HAQ-DI, as defined by MCID > 0.35

Variable	Total sample *N* = 73	Without MCID (ΔHAQ-DI > − 0.35) *n* = 46 (63.0%)	With MCID (ΔHAQ-DI ≤ − 0.35) *n* = 27 (37.0%)		RR (95% CI)		*P*-value
*1.1—Baseline data*							
Female n (%)	43 (58.9)	27 (58.7)	16 (59.3)		1.02 (0.55–1.87)		0.962
**Age, years mean ± SD**	**54.3 ± 9.9**	**56.7 ± 9.1**	**50.3 ± 10.3**		**0.96 (0.94–0.98)**		**0.001**
Time of diagnosis in years*	8 (3–15)	7 (3–16)	8 (2–14)		0.99 (0.95–1.03)		0.493
Symptoms duration in years*	12.0 (5–20)	12.5(5–21)	12.0(6–18)		0.99(0.96–1.02)		0.376
**White ethnicity n (%)**	**65 (89.0)**	**43 (93.5)**	**22 (81.5)**		**0.54 (0.29–1.02)**		**0.058**
CRP in mg/dL*	0.6 (0.4–1.6)	0.4 (0.4–1.7)	0.7 (0.4–1.5)		0.96 (0.8–1.15)		0.630
Patient global VAS in cm*	5.3 (3.6–7.3)	5.0 (3.6–7.1)	5.8 (3.6–7.8)		1.09 (0.97–1.22)		**0.161**
Patient pain VAS in cm*	5.7 (3–8)	5.7 (4.5–8)	6.3 (2.4–7.5)		0.98 (0.89–1.09)		0.694
Physician skin VAS in cm*	1 (0.25–3.4)	1.2 (0.2–3.2)	1 (0.4–5.0)		1.06 (0.96–1.17)		0.241
Patient skin VAS in cm*	4.3 (0.9–7.5)	5.0 (1.2–7.9)	2.4 (0.8–6.3)		0.94 (0.85–1.04)		0.231
Patient fatigue VAS in cm*	6.4 (3.4–8.3)	6.4 (3.5–8.5)	6.5 (3.1–8.3)		1.01 (0.91–1.11)		0.899
MASES*	**0 (0–4)**	**0 (0–3)**	**3 (0–6)**		**1.08 (1.02–1.15)**		**0.013**
TJC 0–68*	2 (0–6)	1 (0–4.5)	2 (0.5–6.5)		1.03 (0.98–1.07)		0.228
SJC 0–66*	0 (0–2)	0 (0–2)	1 (0–2.5)		1.01 (0.93–1.08)		0.888
DAPSA*	16.3 (11.4–22.3)	17.1 (11.0–21.7)	15.2 (11.5–24.8)		1.01 (0.98–1.03)		0.643
DAPSA REM/LDA n (%)	23 (41.1)	4 (38.9)	9 (45.0)		1.17 (0.58–2.37)		0.654
HAQ-DI*	**1.625 (1.0625–2.050)**	**1.375 (0.875–1.906)**	**1.875 (1.250–2.375)**		**1.94 (1.27–2.97)**		**0.002**
MDA 5/7 n (%)	7 (9.9)	6 (13.6)	1 (3.7)		0.35 (0.06–2.21)		0.265
sDMARD use n (%)	54 (75.0)	32 (71.1)	22 (81.5)		1.47(0.65–3.30)		0.355
bDMARD use n (%)	8 (11.0)	6 (13.0)	2 (7.4)		0.65 (0.19–2.24)		0.496
*1.2—Longitudinal data*							
Sustained DAPSA REM/LDA (≥ 12 months) n (%)		41 (56.2)	26 (56.5)	15 (55.6)		0.98 (0.54–1.78)	0.936
**Achieving DAPSA REM/LDA at least once n (%)**		70 (95.9)	45 (97.8)	25 (92.6)		0.54 (0.23–1.27)	**0.155**
ΔDAPSA*		-3.9 (-10.1/ + 2.5)	-3.1 (-9.8/ + 4.6)	-5.0 (-12.9/ + 1.6)		0.98 (0.96 – 1.02)	0.429
**Sustained MDA (≥ 12 months) n (%)**		15 (20.5)	12 (26.1)	3 (11.1)		0.48 (0.17–1.39)	**0.178**
Achieving MDA at least once n (%)		45 (61.6)	28 (60.9)	17 (63.0)		1.06 (0.57–1.97)	0.860
**sDMARD use ≥ 12 months n (%)**		64 (87.7)	38 (82.6)	26 (96.3)		3.66 (0.56–23.7)	**0.175**
bDMARD use ≥ 12 months n (%)		31 (42.5)	17 (37.0)	14 (51.9)		1.46 (0.80–2.65)	0.214

Bold value indiactes statistically significant variables

(*) Variables measured in median (25–75th). MCID, minimal clinically important difference in HAQ-DI, *RR* Relative risk, *CRP* C-reactive protein, *VAS* Visual analogue scale, *MASES* Maastrich Ankylosing Spondylitis Enthesitis Score, *TJC* Tender joint count, *SJC* Swollen joint count, *DAPSA* Disease Activity Index for Psoriatic Arthritis, *HAQ-DI* Health Assessment Questionnaire-Disability Index, *MDA* Minimal disease activity, *REM/LDA* Remission or low disease activity, *ΔDAPSA* Variation of median DAPSA at baseline and at the final patient visit; sDMARD, synthetic disease modifying antirheumatic drugs, *bDMARD* Biological disease modifying antirheumatic drugs.

When analyzing the total sample over 6 years of follow-up, there was no statistically significant improvement of physical function [baseline median (25-75th) HAQ-DI 1.625 (1.0625–2.050), final median HAQ-DI 1.50 (0.75–2.0625), ΔHAQ-DI: − 0.125 (− 0.5 to + 0.375), *p* = 0. 214]. Although there was no statistically significant improvement of physical function when the total sample was analyzed, when patients were analyzed individually, 37% of patients (*n* = 27) showed a clinically meaningful improvement in HAQ-DI (Table 1) despite having long-term PsA.

In the univariate analysis, the variables associated to a MCID in HAQ-DI with a p-value of < 0.20were:age, ethnicity, patient global VAS assessment, median HAQ-DI at baseline, MASES at baseline, sustained MDA, achievement of DAPSA REM/LDA in at least one visit during the follow-up and use of sDMARDs for at least twelve months (Table 1, in bold).

In the multivariate analysis, older age at baseline and white ethnicity were predictors for the non-achievement of the MCID in HAQ-DI [RR 0.96 (95% CI 0.93–0.98, *p* < 0.001) and RR 0.33 (95% CI 0.16–0.67, *p* = 0.002), respectively, while a higher score in HAQ-DI at baseline and the use of sDMARD for more than 12 months were predictors of achievement of the MCID in HAQ-DI [RR 1.71 (95% CI 1.12–2.60, *p* = 0.013) and RR 3.86 (95% CI 1.21–12.3, *p* = 0.022), respectively] (Table 2, in bold).

**Table 2:** Multivariate analysis of predictive factors for patients achieving MCID in HAQ-DI (ΔHAQ-DI ≤ − 0.35)

Variable	RR	95%CI	*P*
Age White ethnicity	**0.96** **0.33**	0.93–0.98 0.16–0.67	** < 0.001** **0.002**
Patient global VASat baseline	0.99	0.89–1.11	0.979
Median HAQ-DI at baseline	**1.71**	1.12–2.60	**0.013**
MASES (enthesitis) at baseline Sustained MDA (≥ 12 months) Use of sDMARD ≥ 12 months Achievement of REM/LDA at least once during follow up Use of sDMARD ≥ 12 months	1.02 0.91 3.22 0.67 **3.86**	0.97–1.08 0.36–2.28 0.79–13.1 0.34–1.33 1.21–12.3	0.454 0.835 0.102 0.248 **0.022**

Bold value indiactes statistically significant variables

The assessment of disease activity over time showed that 56.2% of patients achieved sustained DAPSA REM/LDA and 20.5% achieved sustained MDA in the total sample. The year-by-year longitudinal analysis of function and disease activity is shown in Table 3.

**Table 3:** Information on disease activity, physical function and use of bDMARDs during 6 years of follow-up

Variable	Baseline	Year 1	Year 2	Year 3	Year 4	Year 5	Year 6	*p*
HAQ-DI median (25–75)	**1.625 **^**b**^ (1.031–2.094) n = 72	1.375 (0.625–2.0) n = 52	1.125 (0.594–1.875) n = 50	**1.0 **^**a**^ (0.625–1.843) n = 48	1.125 (0.87–1.812) n = 49	1.25 (0.8 a 1.625) n = 43	1.187 (0.84–1.875) n = 30	**0.015**
DAPSA median (25–75)	**15.9 **^**b**^ (11.4–22.1) n = 57	12.8 (5.6–18.6) n = 47	10.9 (5.8–16.4) n = 50	13.8 (8.0–19.1) n = 45	**11.6 **^**a**^ (7.1–17.1) n = 48	14.1 (8.3–19.9) n = 40	15.0 (9.4–22.1) n = 28	**0.002**
bDMARD use n (%)	**8 **^**a**^ (11,1%) n = 72	**8 **^**a**^ (14,5%) n = 55	**18 **^**b**^ (34%) n = 53	**20 **^**b**^ (41,7%) n = 48	**20 **^**b**^ (40%) n = 50	13 (31%) n = 42	9 (29%) n = 42	** < 0,001**

The p values (highlighted in bold) refer to the statistically significant difference between a and b, compared by Bonferroni test (level of significance 5%). *P* values > 0.05 were removed from the table. HAQ-DI, Health Assessment Questionnaire-Disability Index; DAPSA, Disease Activity Index for PsA. bDMARD, immunobiological disease modifying antirheumatic drugs.

**Fig. 1:**
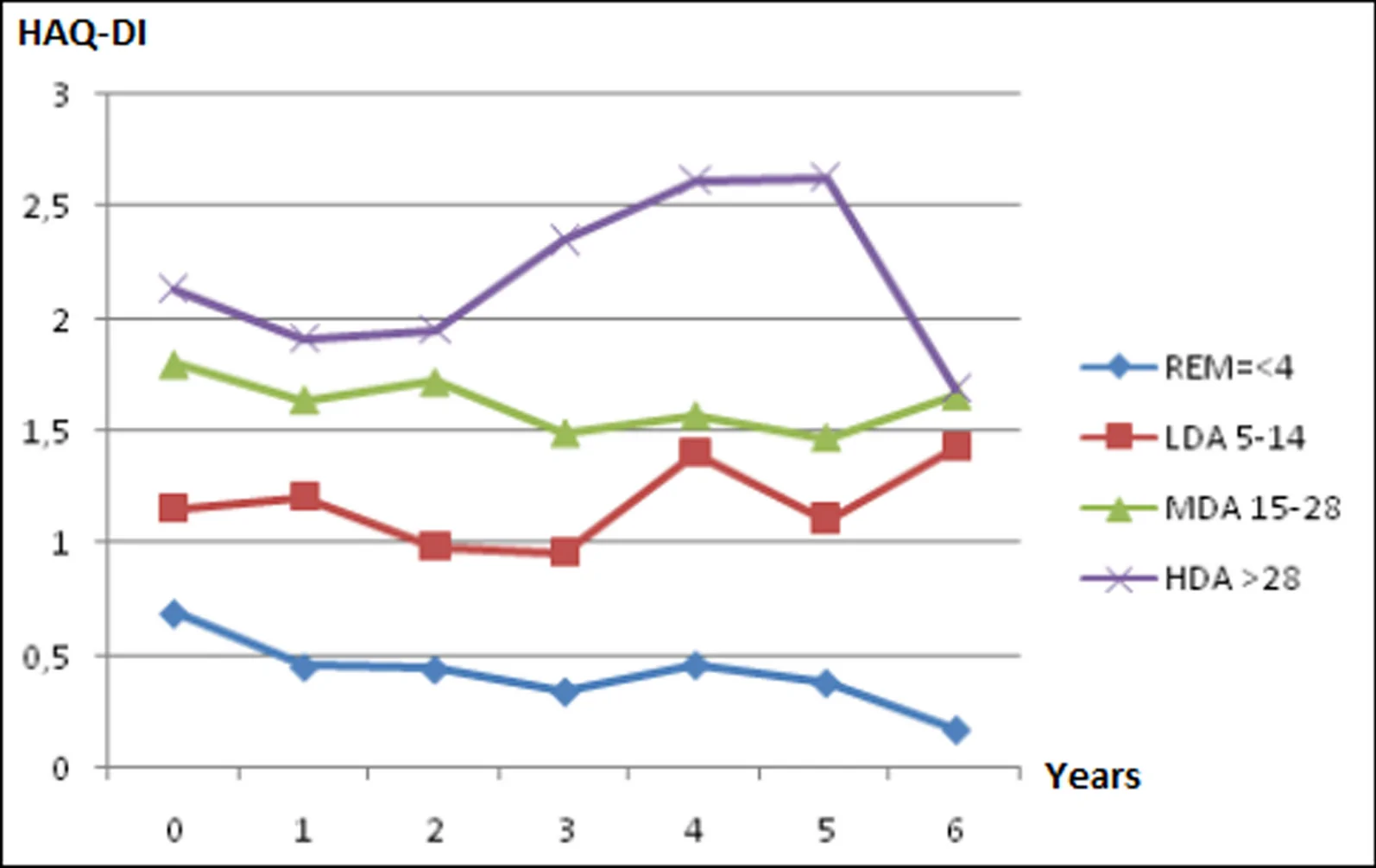
The median HAQ-DI at each year of follow-up divided into four categories according to DAPSA. *HAQ-DI* Health Assessment Questionnaire-Disability Index, *DAPSA* Disease Activity Index for PsA REM: remission, *LDA* Low disease activity, *MDA* Moderate disease activity, *HDA* High disease activity. Patients in remission had the lowest scores on HAQ-DI

A histogram was constructed to display the median HAQ-DI at each year of follow-up according to the four categories of DAPSA (REM, LDA, moderate and high disease activity) (Fig. 1). As expected, the physical function measured by HAQ-DI was determined by the disease activity measured by DAPSA (interaction test *p* < 0.0001). There was an improvement of function in the first three years of follow-up, which was observed in patients in REM, LDA and moderate disease activity by DAPSA, but not in patients with high disease activity. This period of better function was coincidental to the period at which most patients were receiving bDMARDs (Table 3). The only group that showed a sustained reduction in HAQ-DI during the six years was that of patients in DAPSA remission (Fig. 1).

**Fig. 2:**
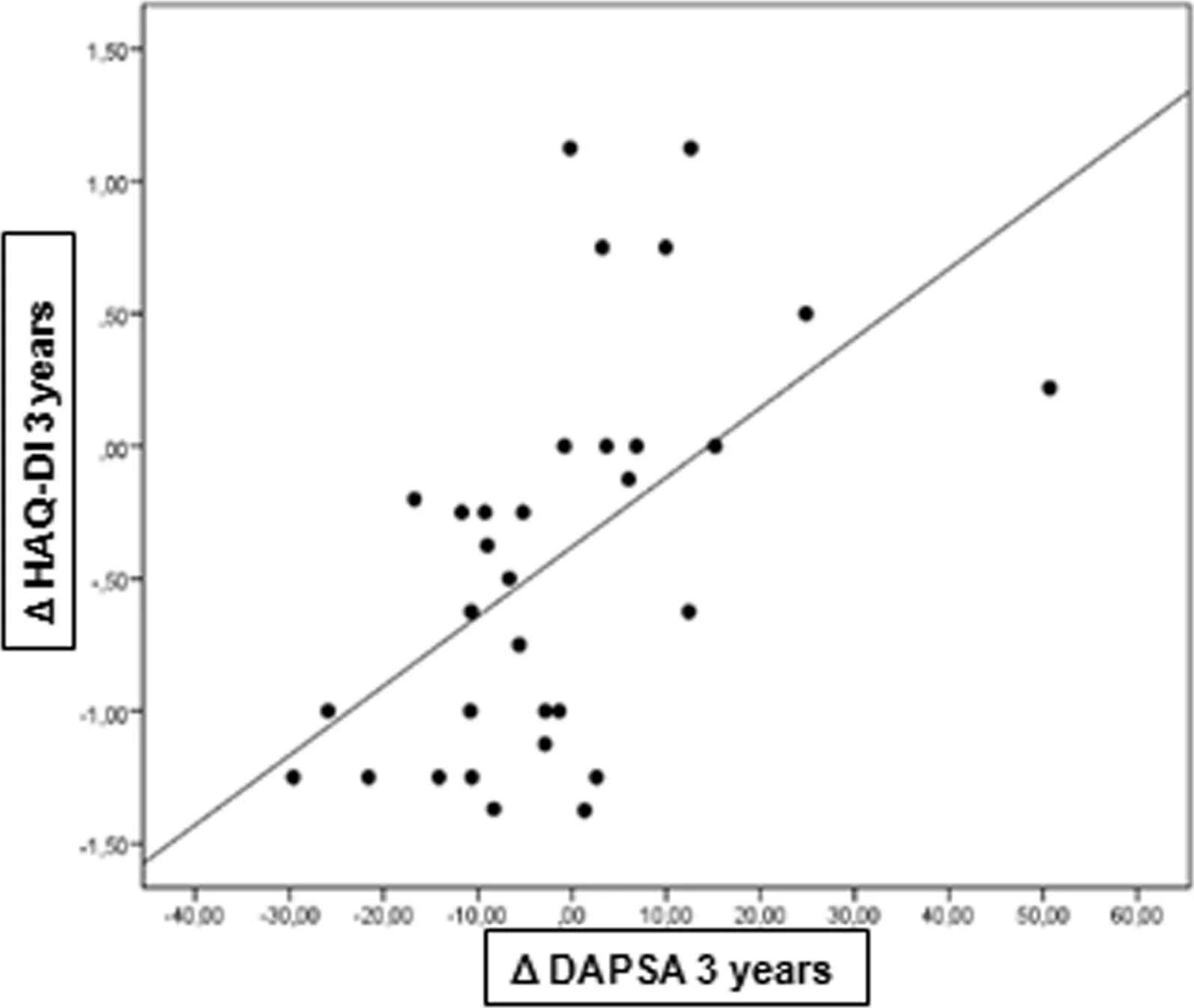
Comparison of the variation of disease activity (ΔDAPSA) and physical function ΔHAQ-DI. *HAQ-DI* Health Assessment Questionnaire-Disability Index, *DAPSA* Disease Activity Index for PsA

The median (25–75th) DAPSA decreased during follow-up [baseline DAPSA 16.3 (11.4–22.3), final DAPSA 12.5 (5.9–18.6),ΔDAPSA − 3.9 (− 10.1/ + 2.5), *p* = 0.019].A moderate and statistically significant correlation was observed between ΔDAPSA and ΔHAQDI (rs = 0.60; *p* < 0.001) (Fig. 2), demonstrating that a reduction in disease activity was associated with an improvement in physical function.

## Discussion

This study was conducted in a real-life setting and led to several interesting results. Firstly, it demonstrated that even patients with long-term PsA can achieve therapeutic targets and obtain an improvement in function; in this cohort, 56.2% of patients achieved sustained DAPSA REM/LDA, 20.5% achieved sustained MDA and 37% obtained a minimum clinically significant improvement in function by HAQ-DI. Secondly, it demonstrated that demographic variables such as younger age and non-white ethnicity, as well as worse functional scores at baseline were predictors of patients achieving a clinically significant improvement in function during follow-up. Finally, we observed that physical function over time was determined by disease activity measured by DAPSA and only the group of patients in DAPSA remission showed values of HAQ-DI constantly lower than 0.5 points, comparable to healthy subjects.

As expected, this improvement was lower in comparison to the clinical trials of patients taking bDMARDs [19, 20], since our cohort includes patients with delayed and difficult access to therapy.

There was a significant improvement in function by HAQ-DI at the first three years of follow-up (Table 2, Fig. 1). However, this tendency was not sustained over the following years, which might be explained by intersubject variability. To our knowledge, other studies evaluating HAQ-DI in PsA present shorter follow-up times (3 to 12 months), which imposes difficulties in comparison to other results [21–23].

Regarding the predictors of MCID, our results were similar to those of previous studies. Other cohorts for assessing physical function measured by HAQ-DI over time in patients with PsA pointed age as an important factor correlated with worse HAQ-DI scores and lower improvement over time [20, 24, 25], showing the influence of comorbidities and established deformities in function. Patients with higher HAQ-DI scores at baseline also had a greater decrease over the follow up, which could be explained by regression towards the mean. The use of sDMARD for more than 12 months was a predictive factor for obtaining MCID, demonstrating the importance of regular treatment in controlling disease activity and improving quality of life.

This cohort showed that white ethnicity was a predictor of not obtaining MCID. Literature describes a higher prevalence of PsA in North America and Northern Europe [26], which is associated with genetic and environmental factors, such as obesity, alcoholism, and the presence of psoriasis. However, ethnicity has not been described as a predictor of improvement in function. Other studies demonstrate that African-Americans with PsA have greater impaired quality of life [27], reinforcing that patients with higher initial HAQ-DI are more likely to achieve MCID.

Several randomized clinical trials comparing biological therapy and placebo have demonstrated MCID in the group using bDMARDs [21, 22, 28]. This direct association was not found in our study. A plausible explanation may be the longer follow-up time and the influence of poor adherence in real life. However, patients taking bDMARDs in our cohort had lower disease activity.

The sample size may have been a limiting factor to find more significant differences between the categories of DAPSA, because the number of patients in each group would not be enough to compare the median HAQ-DI between them. However, it was enough to correlate the variation of DAPSA and HAQ-DI over time, as well as to identify some predictive factors for MCID. The subjectivity of the instruments used must be considered. The DAPSA, MDA and HAQ-DI scores are patient-reported outcome measures and rely on patient assessment about their disease, the presence of pain, tiredness and ability to perform daily activities, which can be influenced by variables not covered in the study, such as the presence of fibromyalgia, mood disorders and other comorbidities [29, 30]. Another limitation is missing data and inter-variability of questionnaire results applied for different physicians. The Rheumatology center minimizes this possible measurement bias by carrying out prior training of the researchers responsible for collecting data.

The strength of this study is that, to our knowledge, it is the first cohort of clinical assistance from the public health service of a developing country, contrasting with the controlled environment of randomized clinical trials. Also, we were able to achieve a longer follow-up time, with an average of 6 years, while most of previous studies evaluating function in PsA have follow-up times from 3 to 12 months [21–23].

## Conclusion

In conclusion, we can postulate that, in the context of developing countries and limited resources, it is worth seeking to achieve lower disease activity scores by DAPSA, regardless of the treatment itself, because they are correlated to better functional outcomes. Higher HAQ-DI scores at baseline, non-white ethnicity and younger age were predictors for achieving a clinically significant improvement in HAQ-DI, reinforcing the importance of early treatment of PsA, before the establishment of deformities.

## Data Availability

Data and materials are available for review.
